# Cancer research: *quo vadis*—to war?

**DOI:** 10.3332/ecancer.2014.ed45

**Published:** 2014-10-21

**Authors:** Denys Wheatley

**Affiliations:** Chairman and Director, BioMedES (www.biomedes.co.uk); Editor *Oncology News* and *Cancer Cell International*; McDonnell Mackie Publishing, Dromore, Co Tyrone, UK

## Abstract

The notion that we should wage all-out war on cancer to break the back of the problem is considered fundamentally untenable because of the inherent nature of the disorder. An alternative is argued on the basis that we can only achieve a greater degree of control through attrition, since only a minority of tumour types might truly be curable in many cases.

## The nature of cancer

Although cancer is an insidious disorder that people strongly wish to eliminate, it does not, like polio, arise from a single causative agent that can be exterminated. Unfortunately, cancer is induced not only by a huge range of different agents depending on the circumstances, but can arise ‘spontaneously’ during a lifetime due to mistakes in the many replicative processes - not just DNA -that inevitably take place within the billions of cells that comprise the human body, many of which are in constant proliferative mode and others that can be brought back into cycle. The word ‘spontaneous’ is in quotes here because in no instance can we be sure that external agents were not involved (e.g. in the diet). There is some difficulty in showing beyond all reasonable doubt that some specific mutation is the underlying cause rather than a variety of unwarranted changes brought about by epigenetic and other events. These intractables have led, nevertheless, to a continuing polarization of views and hypotheses regarding the fundamental changes associated with malignant transformation, with some believing that the somatic mutation ‘theory’ (SMT) is but part of the story [1].

If, as some maintain, cells are always in positive drive, the notion becomes even more plausible that a disturbance - not necessarily a mutational event - such as cells being in an inappropriate environment, results in a marked and persistent change in gene expression that leads to unrestrained division and motility, which might lead to invasive behaviour [2]. This idea is nothing new; the Connheim-Ribbert proposal was of embryonic cell rest, i.e. cells during development not differentiating in the right way at the right time, becoming misplaced and later proliferating to the detriment of the local tissues. It is pertinent at this point to consider the complexity of the carcinogenic process, of how many factors and genes might be involved is the disturbances of proliferative and migratory rates that result in a malignant transformation of a ‘normal cell’. This being a generally accepted premise, it follows that a single mechanism (e.g. a mutation due to a particular environmental carcinogen, such as crocodilite) becomes increasingly unlikely as the number of factors known to be involved increases. I have written on this matter many years ago, since hypotheses in most biological systems are not necessarily exclusive, and indeed this becomes increasingly less probable the greater the number of factors involved and the overall complexity of the phenomenon [3].

To get to grips with cancer, we undoubtedly need to understand all its aspects much more thoroughly, and we should not be complacent in the belief that we have then touched more than the surface of the problem. Research has gone on for well over a century, moving from purely pathological description to experimentation that has given us many useful answers. The question is whether this understanding could ever grow to the point where: (i) we have a ‘general theory’ of cancer, and (ii) we can use this knowledge to prevent its occurrence as far as possible and treat those tumours that nevertheless arise to gain full control over them (hopefully curing the majority of them). Regarding (i), it is highly unlikely that a unifying theory of cancer will ever emerge because of its diversity and complexity mentioned above; to pursue the idea further, especially in isolation, seems a rather fruitless exercise.

## The war strategy

One proposal to move things ahead more rapidly, as espoused by Hanahan [4], is to wage all-out ‘war on cancer’. Researchers worldwide are being called to arms in a concerted campaign, with battle zones on all fronts led by as yet unidentified generals. Nixon declared war on cancer in the early 1970s, believing that throwing enough money at the problem would pay huge dividends within a decade so as to break the back of the problem. Over forty years on, there is only a weak consensus that significant progress has been made [5]. By now, many more billions (nay, trillions) of dollars have been spent on research and developing effective treatments.

Following this new call for action, the question is whether we will see any more significant progress over the next forty years. While we all wish (want) this to happen, it is an unrealistic attitude to expect too much, which may only bring further frustration to many researchers in the field regarding the future direction of cancer research and who will be in charge, a matter already thoroughly discussed by Bagley and Ellis [6]. It is a moot point whether the new CRUK facility in London (The Crick Institute) being directed towards intensive examination of cancer where large groups of researchers each tackling some specific objective might succeed; no one can guarantee faster progress. Without offering 3-year grants that would increase flexibility and the introduction of fresh ideas from young post-docs on the scene, it is less likely (proportionally) to advance our understanding of the many aspects of cancer than disbursing the overall funding more widely. ‘Think tanks’ around the world bringing together top experts with much the same remit have overall failed to generate a plethora of new and now flourishing ideas that have gone into practice.

## Alternatives in this never ending ‘battle’ (challenge)

Is the suggested declaration of war a rational approach? My feeling is that the scenario depicted by Hanahan is inappropriate; there are other scenarios that can more closely resemble the situation than one that leads to an emotional outpouring about ‘battle zones’ and ‘war visions’. My contention, having spent over 50 years researching cancer, is that strategies needed to combat cancer can be better defined by choosing a different analogy, that of it being more akin to terrorism and guerrilla activity. Combatting terrorism is a perennial problem; cancer ***is*** and will continue to be a perennial problem. However, the old adage about any type of warfare applies - ‘know thy enemy’.

The conclusions reached in Weinberg’s very personal article [7] is that the situation has not advanced very significantly within his career in cancer research, which was on genetic mutations and their molecular biological consequences as the underlying rationale. We still do not know much about the enemy, and Weinberg admits the problem is too complex to expect a single concept to provide some basic answers.

We must accept that cancer is and always will be part of our destiny; that mankind will always be developing tumours, and each cancer, like every person, will be unique and will usually require customized treatment. This is a scary proposition not only for its deterministic implication, but because the kind of treatment required cannot realistically be offered to *all* cancer sufferers within any present-day health budget. More to the point, only preventive action has a chance of lessening the load so that fewer cancers that need to be treated will arise in the future. Attempts through screening programmes, vaccines and better control of ‘environmental’ hazards are helping very significantly to reduce the burden, but investment in these aspects has to be sustained so that effective measures are made available worldwide. Educating people about cancer should also be high on the list of priorities.

## Age and cancer

But preventive measures may not fully compensate for the fact that many cancers develop in increasing proportion as longevity increases. Paradoxically, the better our general healthcare is which allows us to live longer, the greater the chance of developing cancer. (One cannot help wondering whether cancer is nature’s way at trying to reduce the proportion of post-reproductive individuals in the human population, a rather facetious Malthusian-based argument!)

War on cancer, as recently presented, is not a quicker way to some panacea or cure. In recent articles [8, 9], I have advocated that *control* is a more rational and realistic objective than an all-out war to find a cure. Quality of life is becoming increasingly seen as the more welcome outcome by patients and oncologists, not just a matter of how many months longer someone can be kept alive (and often has to suffer). We also need to adopt an approach that has the potential to deliver something tangible for anyone like myself who has had to deal with the vicissitudes of actually having had prostate cancer (with the after effects of radiation on the lower bowel). In this regard, any rallying call to war must most definitely ***not bring false hope of a cure*** to cancer sufferers today or at any time in the future.

For these reasons, I believe these articles in question, within a series on ‘Cancer Wars’ in *The Lancet* and the like, given the prominence of some highly respected medical journals, have been unbalanced. They are also poorly argued, lack real insight into the nature and difficulties of cancer and cancer research, fail to consider how research can reasonably (i.e. *practically*) be directed in the future, and give no indication as to how such a war can be implemented financially while retaining the hope of a major breakthrough in the foreseeable future (wars are inordinately expensive, from which no one usually benefits).

Apart from cancer therapy being a problem of attrition, it seems unlikely that putting large numbers of researchers on a major research project, such as the complete sequencing of 100,000 malignant tumours (in a spot-the-difference(s), hopefully in a meaningful rather than an unmeaningful way) will result in significant advances. Progress in science often comes from the isolated and unexpected (serendipitous) incidence. As Szent-György remarked, the important thing is ‘to see what every else sees, but think what no one else thinks’. If 500 scientists are locked into one project, even those who think outside the box will find it difficult to inculcate their new ideas; too much is being or has been committed to large scale projects. Most research today is directed at proving a particular point, research grants being given to those who have already established something that helps move information along *an already predictable pathway* (i.e. low risk). This is quite contrary to the Popperian edict that it is better to test your hypotheses to destruction. Discovery, however, is something being found that was *not* predictable within the corpus of existing knowledge (the received wisdom, e.g. SMT still being seen as an ‘established theory’ rather than a hypothesis). Cancer seems eminently to be one of those problems where inroads and entirely novel approaches will come from some most unlikely places and observations, but research funding is still needed to move on any prior claim to a new and potentially useful advance.

My contention is that cancer has to be seen as a problem at which we just have to keep chipping away, keeping our eyes and minds wide open. It most certainly does not have a single or simple resolution. When ‘breakthroughs’ have been heralded over the years, we find in hindsight that each might have improved some very small facet of this extraordinarily complex condition. After all, understanding of cancer is just one very interesting part of that much bigger problem of ‘what is life?’ [10].

## Conclusion

The prospect of a wide-scale war-like campaign to solve the problems of carcinogenesis and cancer therapy is daunting, with little insight into its enormous complexity, future direction and management in terms of research, let alone the funding it would entail. Cancer, on the contrary, is a perennial problem; every cancer that arises is as unique as the patient, such that each case must be seen as a challenge in itself.
